# Irregular Menses

**Published:** 1887-10

**Authors:** 


### IRREGULAR MENSES.

Catamenia appearing only during the night, *Bovista.* Only during the day, *Causticum.* Increasing during the night, *Ammon. carb.*, and *Zincum.* Not at all in the night, *Causticum.*

Catamenia only in the morning, *Sepia.* During the evening, *Coffea.* Lessening in the afternoon, *Magnes. carb.* Increasing in the afternoon, *Sulphur.* *Pulsatilla*, like *Causticum*, has catamenia during the daytime, but mostly while walking.—*Hering.*

